# Modeled microgravity alters apoptotic gene expression and caspase activity in the squid-vibrio symbiosis

**DOI:** 10.1186/s12866-022-02614-x

**Published:** 2022-08-18

**Authors:** Madeline M. Vroom, Angel Troncoso-Garcia, Alexandrea A. Duscher, Jamie S. Foster

**Affiliations:** Department of Microbiology and Cell Science, Space Life Science Lab, University of Florida, Merritt Island, FL 32953 USA

**Keywords:** Symbiosis, Apoptosis, Caspases, Microgravity

## Abstract

**Background:**

Spaceflight is a novel and profoundly stressful environment for life. One aspect of spaceflight, microgravity, has been shown to perturb animal physiology thereby posing numerous health risks, including dysregulation of normal developmental pathways. Microgravity can also negatively impact the interactions between animals and their microbiomes. However, the effects of microgravity on developmental processes influenced by beneficial microbes, such as apoptosis, remains poorly understood. Here, the binary mutualism between the bobtail squid, *Euprymna scolopes,* and the gram-negative bacterium, *Vibrio fischeri,* was studied under modeled microgravity conditions to elucidate how this unique stressor alters apoptotic cell death induced by beneficial microbes.

**Results:**

Analysis of the host genome and transcriptome revealed a complex network of apoptosis genes affiliated with extrinsic/receptor-mediated and intrinsic/stress-induced apoptosis. Expression of apoptosis genes under modeled microgravity conditions occurred earlier and at high levels compared to gravity controls, in particular the expression of genes encoding initiator and executioner caspases. Functional assays of these apoptotic proteases revealed heightened activity under modeled microgravity; however, these increases could be mitigated using caspase inhibitors.

**Conclusions:**

The outcomes of this study indicated that modeled microgravity alters the expression of both extrinsic and intrinsic apoptosis gene expression and that this process is mediated in part by caspases. Modeled microgravity-associated increases of caspase activity can be pharmacologically inhibited suggesting that perturbations to the normal apoptosis signaling cascade can be mitigated, which may have broader implications for maintaining animal-microbial homeostasis in spaceflight.

**Supplementary Information:**

The online version contains supplementary material available at 10.1186/s12866-022-02614-x.

## Background

Microgravity is a profound source of physiological stress in animals during spaceflight. A reduction in Earth’s gravity can significantly change the shear forces, buoyancy-driven convection, and hydrostatic pressures that organisms experience [1], thus altering critical biological pathways at the cellular, subcellular, and molecular level [2].

One such pathway that can be negatively impacted during spaceflight and microgravity analogs conditions is apoptosis or programmed cell death. Apoptosis is a tightly regulated process that eliminates damaged and unnecessary cells without inflammation and constitutes a critical lever of physiological homeostasis in multicellular organisms [3]. Anomalies in programmed cell death have been attributed to some of the most debilitating health effects of spaceflight, including muscular atrophy, bone demineralization, immune system dysregulation, cardiovascular deconditioning, and visual impairment [4, 5]. Additionally, rodent studies conducted with the hindlimb unloading model, which mimics the reduced weight-bearing of spaceflight through a 30° head-down tilt, have reported higher rates of myonuclear and osteocytic apoptosis during muscular atrophy and bone loss [6, 7]. Other studies have shown that exposure to actual or simulated microgravity conditions can induce apoptosis in the leukocytes of humans and mice, with effects lingering for up to a week post-flight [8, 9]. Endothelial cells (e.g., coronary arteries) also exhibit increased mortality under microgravity conditions [10], which may contribute to cardiovascular deterioration in flight. Collectively, these findings indicate a more intricate understanding of the molecular mechanisms underlying microgravity-induced apoptosis in animals is needed to ensure the continued health and wellbeing of crew members during long-term space missions.

Indeed, dysbiosis is a well-known consequence of spaceflight and there is compelling evidence to suggest that disruptions in an animal’s normal, healthy, microbiome may be related to dysfunctional apoptotic cell death in microgravity [11, 12]. Altered proportions of Firmicutes and Bacteroidetes in the gastrointestinal tract of mice have been associated with reduced apoptosis among colonic epithelial cells in modeled microgravity, thus hindering tissue turnover [13]. Simulated weightlessness has also been shown to induce apoptotic damage in the intestinal mucosal barrier of rats, which is paralleled by compositional changes in the gut microbiome [14].

**Fig. 1:**
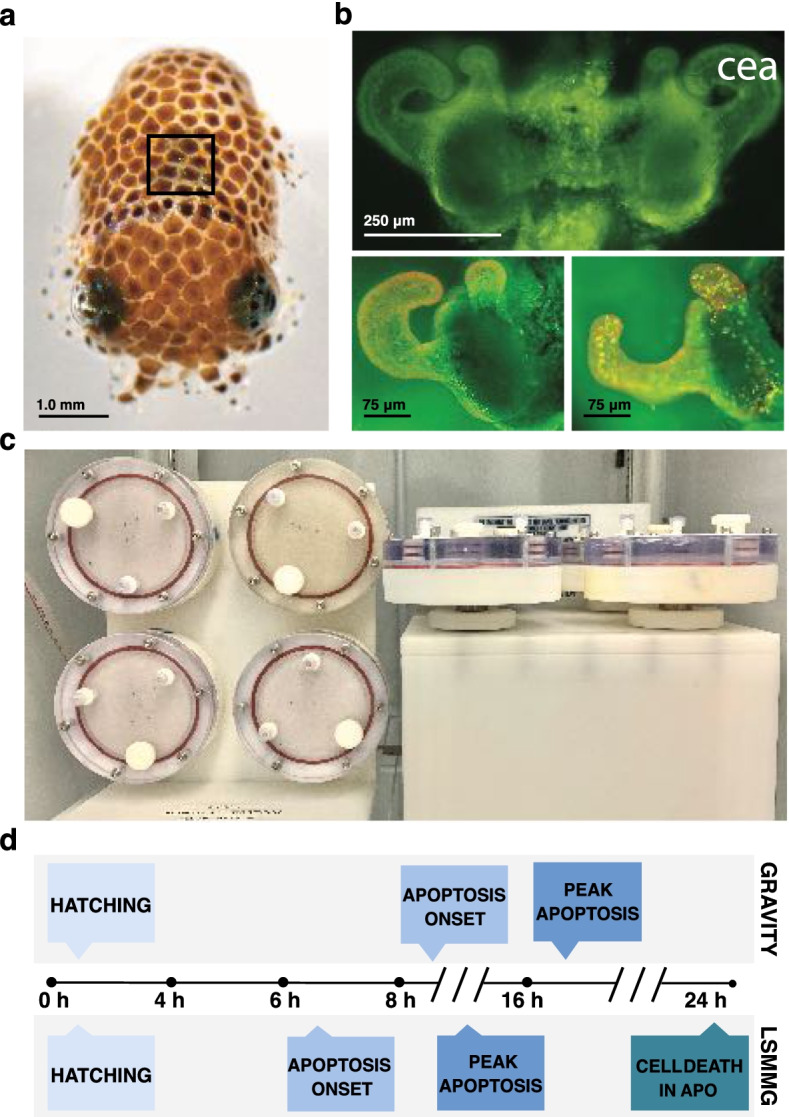
Overview of the host *Euprymna scolopes,* experimental setup, and developmental timeline under different gravitational treatments. **a** Image of *E. scolopes* paralarvae at the time of hatching. The location of the light organ within the host mantle cavity is marked (black box). **b** Fluorescent micrograph showing the bilobed light organ (top) with pronounced fields of ciliated epithelial cells forming distinctive appendage-like structures (cea) extending from either side of the light organ. Light organs stained with acridine orange at the time of hatching (lower left) and during the peak of bacteria-induced apoptosis at 16 h (lower right) show punctate nuclei demarking apoptotic cell death only in those cells exposed to *V. fischeri*. **c** High aspect ratio vessels positioned in the modeled microgravity treatment (left) and gravity (right) control positions. **d** Comparative timeline demonstrating the shift in bacteria-induced apoptosis between gravity and modeled microgravity conditions. The onset and peak of bacteria-induced apoptosis is accelerated in low shear modeled microgravity conditions (LSMMG)

To address these issues, the monospecific mutualism between the Hawaiian bobtail squid, *Euprymna scolopes,* and the gram-negative bacterium *Vibrio fischeri* has emerged as a tractable model system to study the impact of reduced gravity on bacteria-induced changes to host physiology, including apoptosis [15, 16]. *V. fischeri* colonizes a specialized light organ (Fig. 1a-c) in the host squid and produces luminescence that the squid uses at night to conceal its shadow from predators [17]. Shortly after *E. scolopes* hatch, *V. fischeri* are entrained from the surrounding seawater via ciliated epithelial appendages (CEA) that border each lobe of the nascent light organ (Fig. 1b), and in so doing, potentiate colonization. Shortly thereafter, microbe-associated molecular pattern (MAMP) molecules, including lipopolysaccharide (LPS) and tracheal cytotoxin, induce a post-embryonic remodeling event during which apoptosis is widely induced throughout the light organ’s ciliated fields (Fig. 1c), peaking at 16 h [18–20]. Subsequently, in the days following colonization, these structures completely regress [21] and the light organ undergoes further maturation [22].

To simulate microgravity, high aspect ratio vessels (HARVs) have been successfully used with the squid-vibrio symbiosis to generate a low sheared modeled microgravity (LSMMG) environment (Fig. 1c) [15, 16, 23, 24]. Briefly, rotation about a horizontal axis offsets gravitational settling such that the contents of each reactor are maintained in a state of constant suspension under low shear conditions that emulate the quiescent fluid dynamics of low Earth orbit (Fig. 1c). Rotation about a vertical axis, by contrast, allows for sedimentation to occur unperturbed and thus serves as the unit Earth gravity controls (Fig. 1c) [25]. The LSMMG environment produced by this ground-based analog has been used for decades to model microgravity, and results obtained with the HARVs have been shown to parallel the findings of many spaceflight studies [25, 26].

Previously, studies using the squid-vibrio model in LSMMG revealed that the bacteria-induced development of the light organ is accelerated under modeled microgravity conditions (Fig. 1d) [15], however, the mechanisms by which these changes occur are unknown. In this study, we characterize several aspects of the apoptosis genetic machinery in the host squid, examine the differential expression of several of these apoptosis-related genes, and assess the activity and mitigation potential of several initiator and executioner caspases associated with apoptosis under modeled microgravity conditions. The outcomes provide new insight into the mechanisms underlying apoptotic dysregulation in response to microgravity-like stress and its potential impact on the physiology of animals and beneficial microbes during spaceflight.

## Results

### Identification of the extrinsic and intrinsic apoptosis network within the host *Euprymna scolopes*

**Fig. 2:**
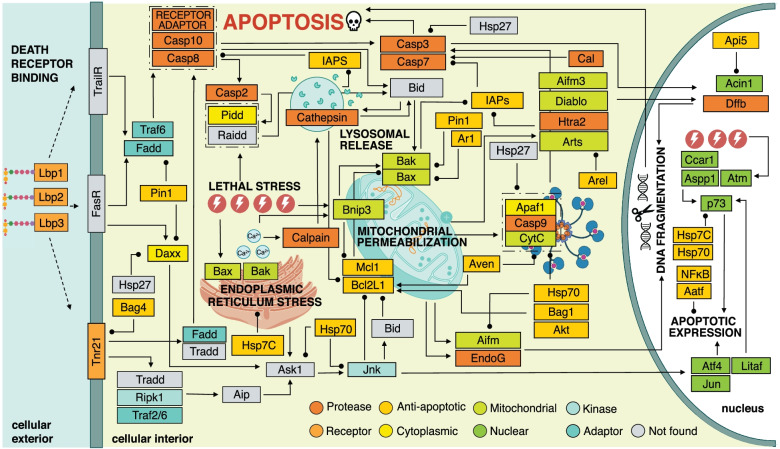
Putative pathways for apoptosis in *Euprymna scolopes.* The candidates identified from the genome and reference transcriptome included effectors of both extrinsic/receptor-mediated, and intrinsic/stress-associated, apoptosis. Representative interactions were mapped by cross referencing the multi-species KEGG pathway for apoptosis (ko04215) with peer-reviewed literature

To elucidate the apoptosis machinery in the bobtail squid, a detailed map of the putative pathways for apoptosis in the squid host was generated by data-mining the reference transcriptome and genome of *E. scolopes* (Fig. 2). This search identified 293 transcripts, representing 137 unique genes, involved in the regulation of apoptosis in the host squid (Table S1; Fig. S1). The resultant network revealed an elaborate web of parallel and hierarchical interactions and pro-and anti-apoptotic effectors that appear to govern extrinsic, receptor-mediated, and intrinsic, stress-induced cell death via transcriptional, translational, and post-translational means (Fig. 2).

For the extrinsic apoptosis network, two tumor necrosis factor receptor superfamily members (Tnfrsf11, Tnfrsf21) and five Tnf-associated factors (Traf 2, 3, 4, 6, and 7) were discovered in *E. scolopes* (Fig. 2, Table S1). The closely related initiator caspases-8 and -10 were also identified, as well as death domain-associated adaptors Fadd, Daxx, and the stress-associated kinase Ripk1. Interestingly, neither Fas ligand nor its corresponding receptor was found in the reference transcriptome despite the presence of components known to operate downstream (e.g., Fadd). Nevertheless, *E. scolopes* was determined to harbor all the effectors required to assemble a complete death-inducing signaling complex (DISC) for extrinsic, receptor-mediated apoptosis, which serves as the activation assembly for extrinsic initiator caspases-10 and -8 [27]. Additionally, transcripts associated with three LPS-binding proteins (Lbps) and the LPS-induced transcription factor, Litaf, which have been previously described in *E. scolopes* [28] were also observed in the putative extrinsic, receptor-mediated network (Fig. 2).

With regards to the intrinsic apoptosis pathway, transcripts encoding both pro-apoptotic (Bnip3, Bcl10, Bcl2L13, Bax, Bak) and anti-apoptotic (Bcl2L1, Mcl1, Ar1) members of the Bcl2 protein family were found in *E. scolopes* (Fig. 2; Table S1). A variety of mitochondrial proteins (i.e., Aifm1, Aifm3, Diablo, Cytc, Htra2) were identified as well as Apaf1 and the intrinsic initiator caspase-9. Thus, all the requisite parts of the apoptosome, which is the activation complex of the stress-induced pathway for cell death were found in the bobtail squid (Fig. 2). However, the Bh3-only protein Bid, which typically represents a critical junction for crosstalk between the intrinsic and extrinsic pathways for apoptosis in animals, was conspicuously absent. Likewise, the presence of atypical initiator caspase-2 was confirmed along with Pidd1, both of which are constituents of the pro-death, p53-inducible, PIDDosome complex that forms in response to DNA damage [29]; however, no evidence of the third member of this assembly, Raidd/Cradd, was found in the bobtail squid reference transcriptome or genome.

In addition to Bcl2-type repressors, transcripts encoding numerous inhibitors of apoptosis proteins (Iaps) were discovered in the host squid. Included among these were Xiap, Diap2, three Baculoviral Iap-repeat containing proteins (Birc7b, Birc3, and Birc6), three Bcl2-associated athanogene regulators (Bag 1, 4, and 6), cell death regulator Aven, the peptidyl isomerase Pin1, as well as heat shock proteins Hsp90 and Hsp7c (Table S1). Further examination revealed the presence of numerous cathepsin (B, K, L, L1, L2, Z) and calpain (1–3, 5, 7–9, 11, B, D) proteases, several of which have been previously reported in *E. scolopes* [30]. The cathepsins recovered from the reference transcriptome were primarily cysteine-dependent, except for cathepsin B, which is a serine protease. Intriguingly, neither aspartic acid-type cathepsins (D and E) were found in the host squid.

Analysis of the *E. scolopes* reference transcriptome revealed a variety of nucleus-localizing proteins affiliated with apoptosis (Table S1). Broadly, this included transcription factors (i.e., Aatf, Atf4, Jun, E2f, Hox, Irf, Litaf, NF-κB, p73), p53-type transactivators (i.e., Aspp1, Ccar1), and apoptotic mediators of chromatin condensation and DNA fragmentation (i.e., Acin1, Dffb/Cad, EndoG) (Table S1). Transcripts encoding numerous regulators of apoptotic translation were likewise present in the squid, most notably the eukaryotic initiation factors Eif2, Eif3f, Eif3j, Eif4b, and Eif4e (Table S1). Interestingly, however, no transactivating factors of internal ribosomal entry site (IRES)-mediated translation were present in the transcriptome of *E. scolopes*, and the nuclear lamin protease and executioner caspase, caspase-6, was not observed.

### Extrinsic and intrinsic apoptosis gene expression under LSMMG conditions

**Fig. 3:**
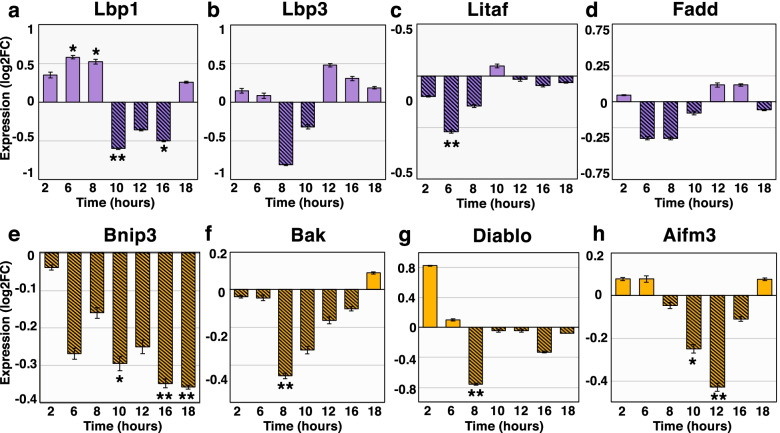
Extrinsic and intrinsic gene expression in symbiotic hatchlings in gravity controls relative to LSMMG. Expression of extrinsic, receptor-mediated apoptosis genes **a** Lbp1, **b** Lbp3, **c** Litaf, and **d** Fadd. Expression of intrinsic, stress-induced apoptosis genes **e** Bnip3, **f** Bak, **g** Diablo, and **h** Aifm3. Expression is conveyed as log2 fold-change (log2FC). Positive log2FC values denote higher expression in the gravity control group (solid bars). Negative log2FC values indicate up expression in LSMMG (hatched bars). Error bars are the standard error of the mean. Asterisks denote significant differences between the conditions (* = *p* ≤ 0.10, ** = *p* ≤ 0.05)

**Fig. 4:**
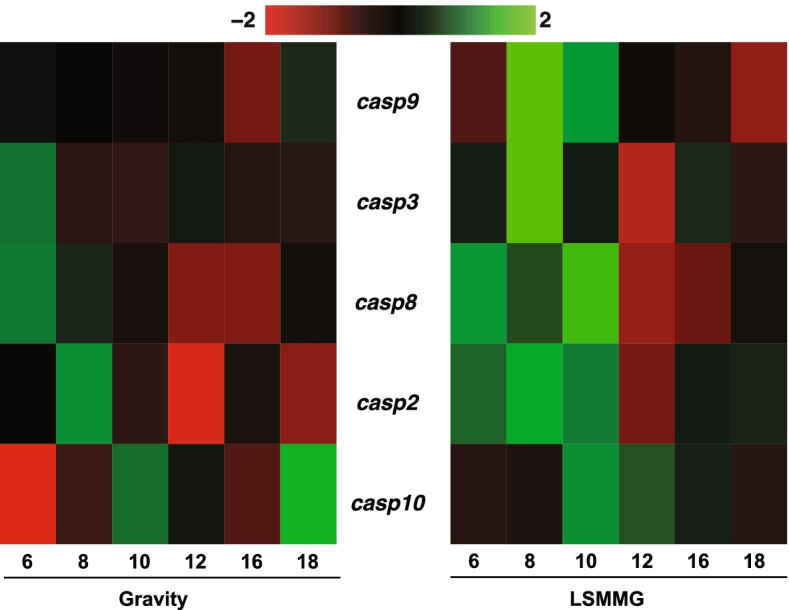
Apoptotic caspase expression in symbiotic hatchlings under gravity and low shear modeled microgravity (LSMMG) conditions. Heatmaps representing the transcriptional expression of pro-death caspases -2, -3, -8, -9, and -10 genes in gravity (left) and LSMMG (right) conditions. Per the color scale, red indicates a negative Z-score and lower-than-average expression, whereas green signifies a positive Z-score and higher-than-average expression

To ascertain the impact of modeled microgravity on the light organ cell death event in the host animal, 41 apoptosis-related genes identified in the reference transcriptome were selected for gene expression analysis using the NanoString nCounter assay (Table 1). These target genes were representative of key components of the apoptosis machinery depicted in Fig. 2. The expression of these target transcripts was quantified over time in hatchling paralarvae incubated within the HARVs under LSMMG and gravity control conditions in the presence and absence of the symbiotic bacterium *V. fischeri* ES114. Overall, the NanoString results indicated that there was an increase in the transcription of genes associated with both receptor-mediated and stress-induced apoptosis under LSMMG conditions compared to gravity controls (Fig. 3, 4).

**Table 1:** NanoString CodeSet for apoptosis genes in *Euprymna scolopes*

Protein	Functional description
Acin1	Mediates apoptotic chromatin condensation following caspase 3 activation without fragmentation
Aifm1	Contributes to caspase-independent apoptosis, also activates executioner caspases 7
Aifm3	Implicated in intrinsic, caspase-dependent, apoptosis via reduced mitochondrial membrane potential
Apaf1	Forms the apoptosome complex with cytochrome C
Api5^a^	Anti-apoptotic factor that inhibits E2f1-induced apoptosis
Arts	Regulates cytoskeletal organization and is required for Tgf-β induced apoptosis
Aspp1	Enhances the transactivation and DNA-binding activity of p53 at the promoters of pro-apoptotic genes
Atm	Senses DNA damage and activates checkpoint signaling in response to apoptotic or genotoxic stressors
Aven	Inhibits Apaf1-dependent intrinsic apoptosis
Bcl2L1	Blocks caspase activation and the release of cytochrome C from the mitochondria. BclXL isoform
Bag1	Strengthens anti-apoptotic Bcl2 repression, inhibits pro-death PP1R15 phosphatase
Bag4	Prevents constitutive signaling via Tnf superfamily receptor member 1A
Bak	Forms pores in the mitochondrial membrane via oligomerization in response to intracellular stress
Bax	Upon activation results in cytochrome C release and intrinsic apoptosis
Birc6	Targets caspases 3, 7, and 9 as well as mitochondrial-derived Diablo/Smac for proteasome degradation
Bnip3	Overcomes Bcl2-type suppression of pro-death effectors Bax/Bak. Induced by hypoxia-dependent signaling
Casp2	Activated in the DISC via caspase 8, the intrinsic p53-induced PIDDosome, and is cleaved by caspase 3
Casp3	Pro-apoptotic executioner and targets structural proteins and activates effectors to assist in cell breakdown
Casp7^a^	Pro-apoptotic executioner and targets structural proteins and activates effectors to assist in cell breakdown
Casp8	Pro-apoptotic initiator and activated during extrinsic, receptor-mediated, apoptosis via DISC assembly
Casp9	Pro-apoptotic initiator and activated via the apoptosome in response to fatal levels of intracellular stress
Casp10	Pro-apoptotic initiator and activated during extrinsic, receptor-mediated, apoptosis via DISC assembly
CatL^a^	Lysosomal cysteine protease and degrades pro-death CatD, protects against neuronal apoptosis
Daxx^a^	Proposed to mediate apoptosis in response to Fas death receptor stimulation
Diablo	Promotes caspase-dependent apoptosis and suppresses inhibitors of apoptosis after mitochondrial release
Diap2	Inhibitor that inactivates effector caspase Drice (caspase 3 homolog) via ubiquitination
Fadd	Adaptor protein that recruits and activates caspases 8 and 10 in response to Tnfr stimulation
Hsp7c	Up-expression correlates with reduced apoptosis. Binds LPS. High levels inhibit p53 activation
Hsp90	Inducible molecular chaperone that stabilizes Akt kinase, which targets caspase 9 to inhibit apoptosis
Jnk	Phosphorylates transcription factors in response to radiation, including Jun, and promotes apoptosis
Jun	Demonstrated role in promoting apoptosis, antagonized by Bcl2 inhibitor
Lbp1	Binds LPS by the lipid A region. Plays a role in establishing the light organ symbiosis
Lbp2	Binds gram-negative LPS by the lipid A region
Lbp3	Binds gram-negative LPS by the lipid A region
Litaf	p53 inducible, promotes Tnf-α transcription in response to LPS. Inhibits anti-apoptotic Bcl6 and BclXL
Mcl1	Promotes cell survival and inhibits intrinsic apoptosis by blocking Bak/Bax
Pin1	Promotes cellular survival and inhibits pro-apoptotic factors including Bak, Bax, Fadd, and Daxx
RipK1	Activates pro-death Jnk cascade in response to Tnf-α. Also promotes cell survival via NF-κB activation
Traf2	Promotes p53-dependent apoptosis via Jnk activation. Regulates NF-κB
Traf6	Activates NF-κB and contributes to apoptosis through Ask1-dependent Jnk activation
Xiap^a^	Inhibitor of apoptosis that targets proteins to proteasome for degradation via ubiquitination

^a^ Indicates the genes for which the nCounter probes failed

With regards to effectors of extrinsic, receptor-mediated, apoptosis, the LPS binding proteins 1 and 3, along with the endotoxin-induced transcription factor Litaf were significantly up-expressed in modeled microgravity between 6 and 10 h post-hatching (Fig. 3). Likewise, transcripts encoding the adaptor protein Fadd were more abundant during the early hours of development in LSMMG; however, these results were not statistically significant from the gravity controls (Fig. 3).

A similar trend was observed in the NanoString results for genes affiliated with intrinsic, stress-induced apoptosis. The Bh3-only protein Bnip3, membrane permeabilizer Bak, Diablo, and caspase-dependent Aifm3 were significantly up expressed in LSMMG between 8 and 12 h compared to gravity controls (Fig. 3). Bnip3, which overcomes Bcl-2 type suppression of pro-death effectors Bax/Bak, increased overtime under LSMMG peaking at 16 and 18 h in LSMMG compared to the unit gravity controls (Fig. 3). Bnip3 is known to be induced under hypoxic conditions, however, measurements of the dissolved oxygen content were consistent across all treatments at 24 h revealing no difference in oxygen availability in the HARVs (Fig. S2).

### Expression of pro-death caspases in host animal under LSMMG conditions

The identification of caspase-2, -3, -7, -8, -9, and -10 transcripts in the reference transcriptome suggested the presence of canonical pathways for extrinsic and intrinsic apoptosis in *E. scolopes* (Fig. 4). Additionally, caspase-driven apoptosis has a known role in the bacteria-induced development of the light organ, given that treatment with the pan-caspase inhibitor Z-VAD-FMK has been shown to reduce the number of dying cells observed at 24 h post-inoculation [30]*.* These results suggested that caspase regulation, either at the level of mRNA and/or protein, was a contributing factor to the accelerated developmental timeline of apoptosis in the host light organ under LSMMG conditions (Fig. 1d).

NanoString analysis was used to examine the differential gene expression of the putative initiator (i.e., *casp2*, *casp8*, *casp9*, and *casp10*) and executioner (i.e., *casp3*) caspase genes in symbiotic and aposymbiotic squid under microgravity and gravity conditions. Results revealed that under LSMMG conditions there was a pronounced increase in the pro-death caspase transcripts between 6 and 10 h post-colonization by *V. fischeri* (Fig. 4), which aligns with early-onset apoptosis previously observed in paralarvae squid exposed to modeled microgravity [15]. Aposymbiotic control animals that were not exposed to *V. fischeri* showed no differential expression under LSMMG or gravity conditions at these same time points (Fig. S3).

### The architecture of caspase domain structure in *Euprymna scolopes*

**Fig. 5:**
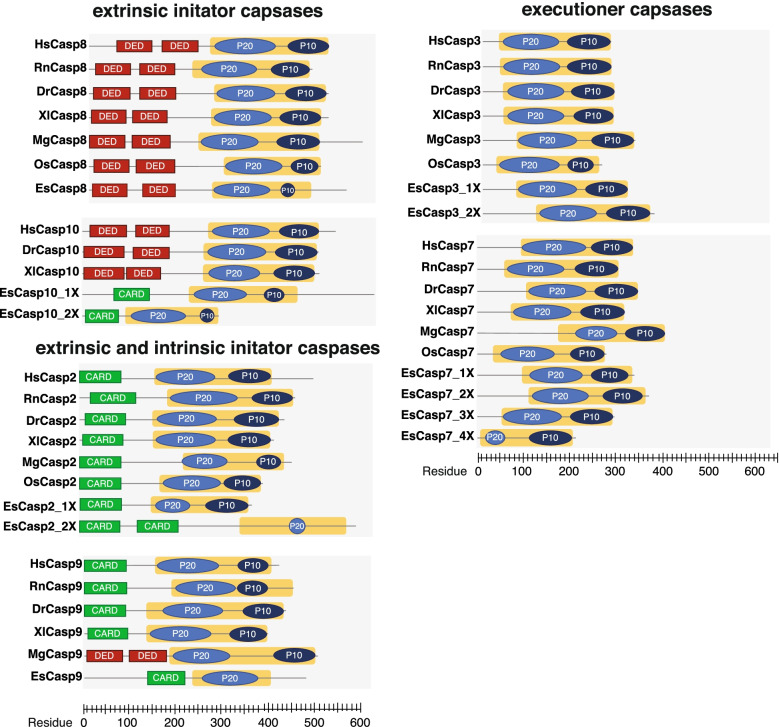
Protein sequence-based analysis of modular domain architecture for the initiator and executioner caspases of *E. scolopes*, *H. sapiens*, *R. norvegicus*, *D. rerio*, *X. laevis*, *M. galloprovincialis*, and *O. sinensis*. The X-axis indicates residue position in the primary peptide sequence. Red boxes represent death effector domains (DED) whereas green boxes are caspase activation and recruitment domains (CARD). The 45 kDa CASc precursor is illustrated by a yellow box, with the resulting p20 and p10 subunits shaded medium and dark blue, respectively. Variants of squid caspase enzymes are indicated by the notation *n*X where *n* is the isoform number

To evaluate the extent of similarity between the *E. scolopes* caspase (EsCasp) enzymes and homologs in other animals, the domain architecture of EsCasps was mapped and compared to sequences derived from *Homo sapiens, Rattus norvegicus*, *Danio rerio*, and *Xenopus laevis* (Fig. 5). Additionally, the Mediterranean mussel, *Mytilus galloprovincialis*, and the East Asian octopus, *Octopus sinensis*, were included in the analysis to distinguish lineage-specific modifications within the Mollusca phylum and/or among cephalopods (Fig. 5, Additional File 3). Domain analysis via SMART, InterProScan, and the NCBI Conserved Domain Database, as described in the Methods, revealed that the architecture of caspases-2, -3, -7, -8, and -9 in *E. scolopes* is largely consistent with that of other animals, including both vertebrates and invertebrates alike (Fig. 5). Typically, the EsCasps ranged from 327 to 634 residues in length and weighed between 41.4 and 72.1 kDa (Table 2). The main exception to this was EsCasp10_2X, which was only 297 amino acids long and had a molecular weight of 34.4 kDa (Table 2).

**Table 2:** Initiator and executioner caspases in Euprymna scolopes

Name	Type	Catalytic pentapeptide	Molecular weight (kDa)	Length	pI	BLASTx E-score	topBLASTx Organism
EsCasp10_1X	Initiator	QACQP	72.1	634	5.83	9.28e-16	Caspase 10 Homo sapiens
EsCasp10_2X	Initiator	QACQP	34.4	297	4.69	7.95e-17	Caspase 10 Homo sapiens
EsCasp8	Initiator	QACQG	63.1	560	7.76	2.78e-57	Caspase 8 Rattus norvegicus
EsCasp2_1X	Initiator	QANSV	67.7	591	5.28	3.58e-08	Caspase 2 Mus musculus
EsCasp2_2X	Initiator	QACRS	41.4	368	5.67	4e-25	Caspase 2 Mus musculus
EsCasp9	Initiator	QACQP	46.8	481	4.77	3.44e-13	Caspase 9 Homo sapiens
EsCasp7_1X	Executioner	QSCRG	38.4	337	8.62	89e-58	Caspase 7 Mesocricetus auratus
EsCasp7_2X	Executioner	QACRG	41.5	370	5.13	6.09e-70	Caspase 7 Homo sapiens
EsCasp7_3X	Executioner	QACRG	33.9	296	6.71	1.39e-71	Caspase 7 Mus musculus
EsCasp7_4X	Executioner	QACRG	23.9	213	4.75	7.03e-56	Caspase 7 Mus musculus
EsCasp3_1X	Executioner	QACRG	35.3	313	6.97	2.63e-65	Caspase 3 Bos taurus
EsCasp3_2X	Executioner	QACRG	41.2	365	6.8	4.52e-72	Caspase 3 Mus musculus

Several EsCasps exhibited key structural elements characteristic of caspases in other animals (Fig. 5). For example, the extrinsic initiator caspase-8 of *E. scolopes* (EsCasp8) exhibited dual twin death effector domains (DED) at the N-terminus, which is characteristic of both vertebrates and invertebrate caspase-8 proteins; however, this dual DED domain was missing in the initiator EsCasp10 isoforms when compared to vertebrates *H. sapiens, D. rerio,* and *X. laevis* (Fig. 5). EsCasp10 did harbor a singular caspase activation and recruitment domain (CARD), suggesting a potential misannotation.

The CARD domain was also observed in EsCasp2 and -9 initiator caspases, which is typical of most vertebrates and invertebrates (Fig. 5). Interestingly, caspase-9 in *M. galloprovincialis* (MgCasp9) exhibited dual DED in place of the traditional CARD, suggesting a potential misannotation of this protein in *M. galloprovincialis*. In all sequences examined, a carboxyl-terminal catalytic (CASc) domain was present (Fig. 5, yellow box). The CASc is a 45 kDa precursor that is cleaved during activation to yield the p20 and p10 subunits. Intriguingly, despite the presence of a complete CASc, analysis of EsCasp2_2x and EsCasp9 revealed the smaller p10 subunit was not detected in these two isoforms.

Executioner caspases-3 and -7 in animals typically lack pro-domains and are therefore shorter as a result (Fig. 5). The executioner caspases of *E. scolopes* (EsCasp3 and -7) varied from 213 to 370 residues in length and had a molecular weight ranging from 23.9 to 41.5 kDa (Table 2; Table S2), thus were comparable to both the vertebrate and invertebrate species targeted in this analysis. Most EsCasp3 and -7 isoforms exhibited a conserved QACRG pentapeptide in the catalytic region or equivalent (Table 2).

### Phylogenetic analysis of host squid caspases

**Fig. 6:**
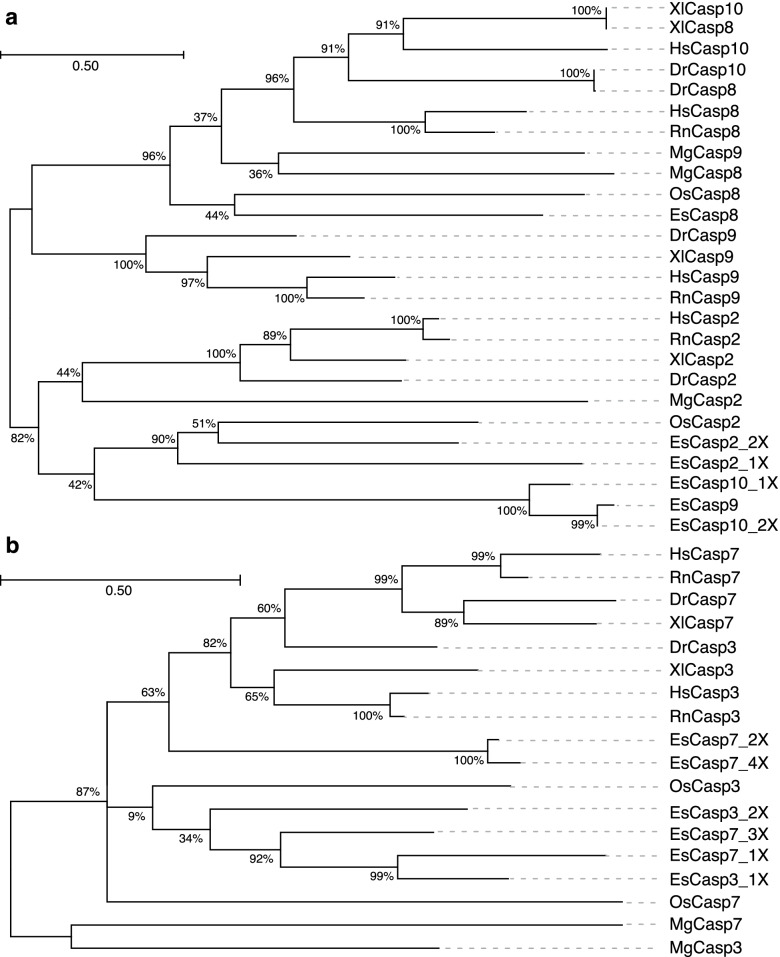
Maximum likelihood phylogenetic analysis of the caspase enzymes in *E. scolopes, H. sapiens*, *R. norvegicus*, *D. rerio*, *X. laevis*, *M. galloprovincialis*, and *O. sinensis*. a Unrooted phylogenetic tree for initiator caspase-2, -8, -9, and -10*.* b Unrooted phylogenetic tree for executioner caspase-3 and -7. Both trees were generated assuming the WAG model of amino acid substitution, with 1000 bootstrap iterations, in MEGA X. Branch support values are expressed as percentages. Sequences are labeled with the first letter of the corresponding genus and species (e.g., *Euprymna scolopes, Es*)

Maximum likelihood analysis of the initiator caspases revealed two distinctive clusters primarily consisting of caspases-8, -9 and -10, and caspase-2, respectively (Fig. 6a), that were reflective of the function of whether the initiator caspases were activated by extrinsic or intrinsic triggers. EsCasp8 clustered with all other caspase-8 proteases sourced from NCBI but was closest to proteases from *O. sinensis.* Similarly, EsCasp2 also branched closely with both invertebrate and vertebrate caspase-2 initiators.

Unexpectedly, EsCasp9 clustered with the two sequences that were likely misannotated as isoforms of caspase-10. Although the monophyly of the group is indeterminate due to the low outer branch support value (42%), these sequences share several distinguishing features that suggest they may represent a group of caspase-9-like initiators that is specific to *Euprymna* (Fig. 6a)*.* First, the domain organization of EsCasp9, EsCasp10_1X, and EsCasp10_2X emulates that of canonical caspases -2 and -9, with an N-terminal CARD domain and a C-terminal CASc region (Fig. 5). Second, although the sequences of this group exhibit 94 to 95% identity with one another, their percent identity scores with the other initiator caspases are very low, between 15 and 29%, which indicates they may have distinct structural features (Fig. S4). Third, EsCasp9, EsCasp10_1X, and EsCasp10_2X all exhibit a conserved “QACQP” catalytic pentapeptide (Table 2). Caspases, uniformly, possess a QACXG motif in the active site, with the middle position specifying the catalytic cysteine residue [31]. Given its crucial importance to the protease activity of caspases, variations within this pentapeptide are limited and may denote similar functions.

The maximum likelihood estimation of executioner caspases formed two several distinctive groups primarily separated into vertebrate and invertebrate clusters (Fig. 6b). For example, the vertebrate caspases-7 and -3 from *H. sapiens, R. norvegicus, D. rerio*, and *X. laevis* formed distinctive groupings, that excluded most of the molluscan isoforms. Interestingly, two caspase-7 isoforms from *E. scolopes* (EsCasp7_2X and EsCasp7_4X) had a higher association with the vertebrate executioner caspases than the other molluscan orthologs for caspase-3 and -7 (Fig. 6b). This phylogeny may allude to a greater degree of differentiation, or perhaps expansion, among the executioner caspases of the squid, which has previously been documented in bivalves [32, 33]. However, the percent identity among the executioners was 47% which makes it difficult to discern whether these sequences represent isoforms of the same gene(s) or an expansion of caspases-3 and -7 in *E. scolopes* (Fig. S4).

Overall, there appeared to be a relatively high degree of similarity among the executioner caspases of *E. scolopes*. However, the close affiliation of EsCasp7_2X and EsCasp7_4X with vertebral orthologs suggests the presence of at least two distinct executioner-type caspases in the squid. Unsurprisingly, both the initiator and executioner caspases of *E. scolopes* were most closely related to *O. sinensis,* and to a lesser extent, *M. galloprovincialis*, which is consistent with their categorization as both soft-bodied cephalopods and, more broadly, mollusks.

### Caspase activity and mitigation during the onset of symbiosis and light organ morphogenesis under LSMMG and gravity conditions

**Fig. 7:**
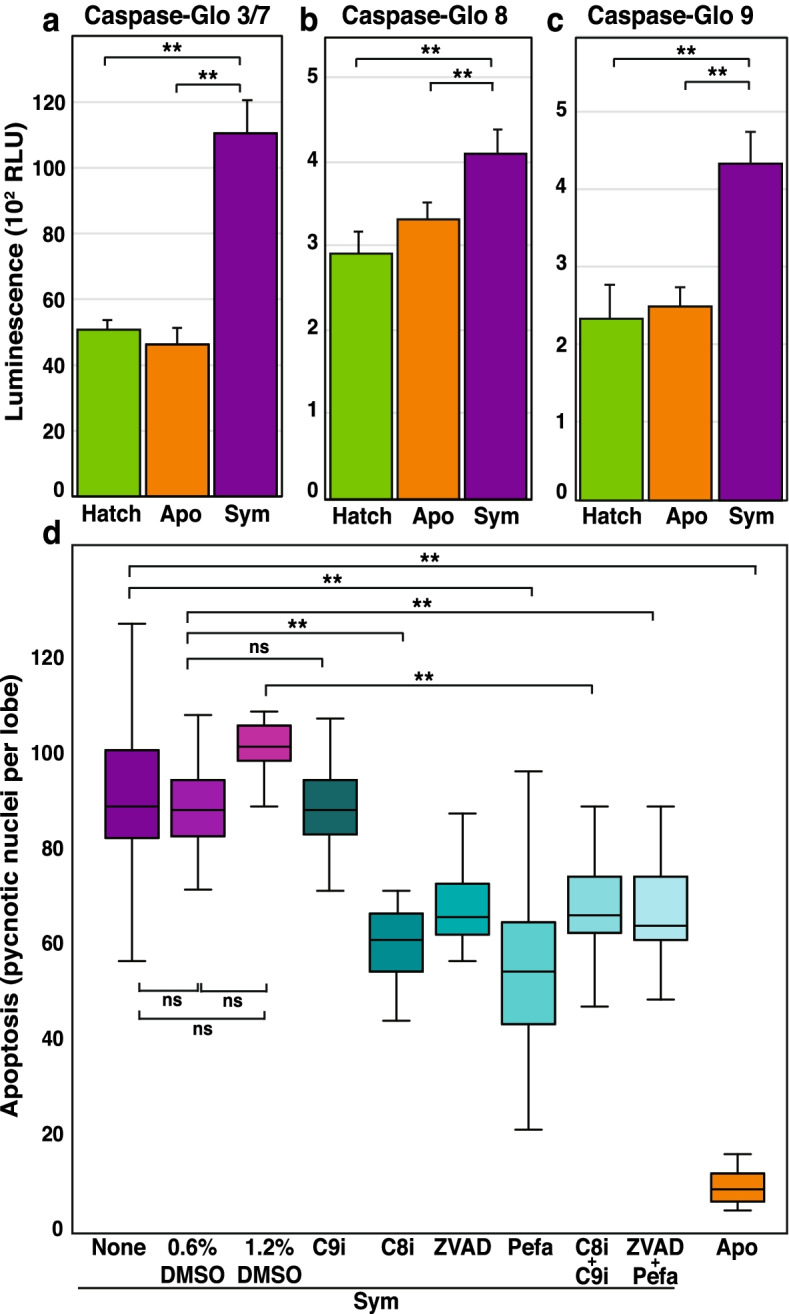
Caspase activity and protease inhibition during bacteria-induced apoptosis in the normal squid-vibrio symbiosis. The activity of a executioner caspase-3/-7, b extrinsic initiator caspase-8, and c intrinsic initiator caspase-9 was measured in the light organs of hatchling, 16 h aposymbiotic (apo), and 16 h symbiotic (sym) squid. d Apoptosis was quantified at 16 h after protease inhibitor treatment including pan-caspase inhibitor z-VAD-FMK (ZVAD), the caspase 8 inhibitor Ac-IETD-CHO (C8i), caspase 9 inhibitor Ac-LEHD-CMK (C9i), Pefabloc (Pefa) and dimethyl sulfoxide (DMSO) controls. Error bars are the standard error of the mean. Asterisks denote significant differences between the datasets test (* = *p* ≤ 0.05, ** = *p* ≤ 0.01). Comparisons that were not significant are labeled “ns”

The activities of executioner caspases-3/7, extrinsic initiator caspase-8, and intrinsic initiator caspase-9 were quantified in host animals in the presence and absence of the symbiont *V. fischeri* using Caspase Glo activity kits. Note, due to the similar mechanisms of activity, the kits could not resolve between caspase-3 and caspase-7 activities. All caspases tested were significantly more active in symbiotic animals at 16 h post-colonization, the peak of cell death [19], compared to 16 h aposymbiotic controls and newly hatched paralarvae (Fig. 7). In all three treatments, there was a 26-fold increase in executioner caspase activity (Fig. 7a) compared to the extrinsic and intrinsic initiators (Fig. 7b, c).

To test whether the caspases could be inhibited in the host animals, pre-treatment with inhibitors targeting different caspases was used. Treatment with the caspase-8 inhibitor Ac-IETD-CHO, the pan-caspase inhibitor Z-VAD-FMK, and the serine protease inhibitor Pefabloc for two hours prior to the start of the symbiosis significantly reduced apoptotic cell levels at 16 h in symbiotic hatchlings compared to untreated symbiotic controls but did not completely inhibit apoptosis (Fig. 7d). Interestingly, although the Caspase-Glo assays indicated caspase-9 was active during the apex of cell death (Fig. 7c), treatment with the caspase-9 inhibitor Ac-LEHD-CMK did not reduce the level of pycnotic nuclei observed at 16 h relative to the untreated or DMSO-treated controls (Fig. d). Additionally, the coupling of different inhibitors showed no additive effects (Fig. 7d). The colonization of the host squid did not appear to be affected as symbiotic animals exhibited normal luminescence levels throughout the experimental treatments.

**Fig. 8:**
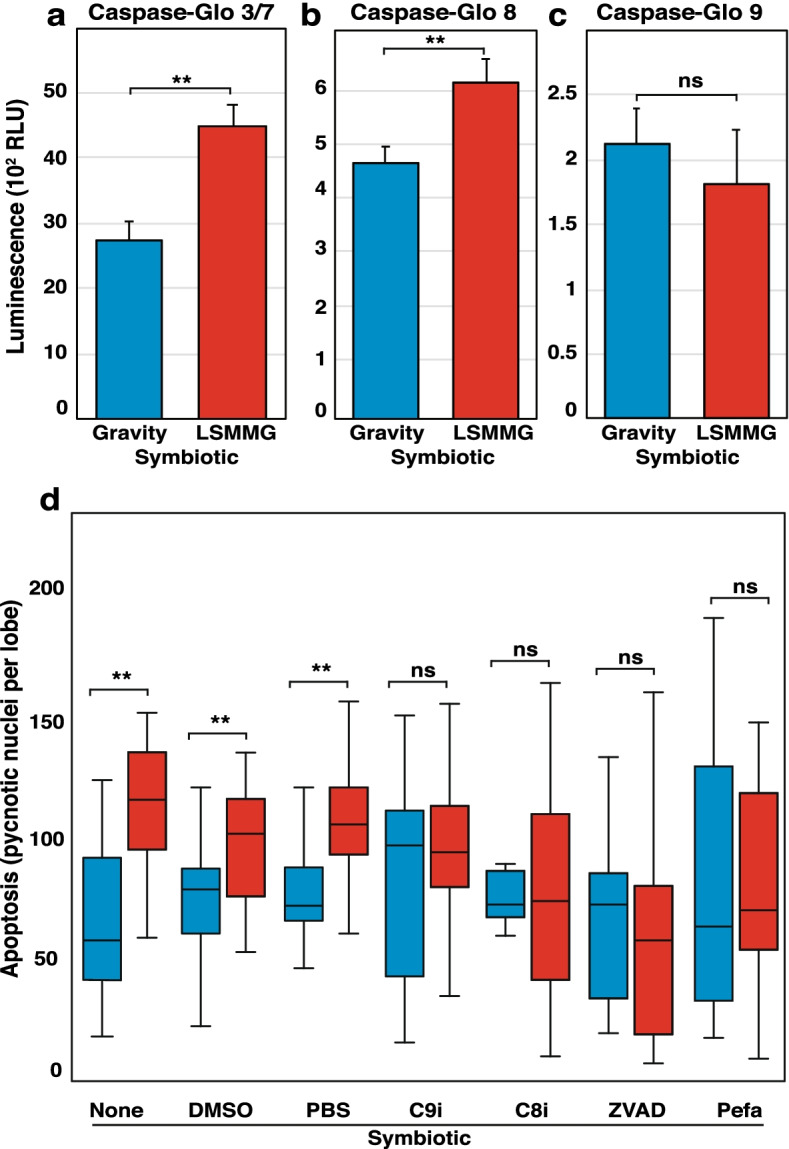
Caspase activity and protease inhibition during light organ apoptosis in gravity and LSMMG conditions at 10 h post-inoculation. The activity of a executioner caspases 3/7, b extrinsic initiator caspase-8, and c intrinsic initiator caspase-9 was measured in the light organ of symbiotic (sym) hatchlings at 10 h in gravity (blue) and modeled microgravity (red). d Apoptosis was quantified in symbiotic hatchlings at 10 h in both conditions following protease inhibitor treatment including pan-caspase inhibitor z-VAD-FMK (ZVAD), the caspase 8 inhibitor Ac-IETD-CHO (C8i), caspase 9 inhibitor Ac-LEHD-CMK (C9i), Pefabloc (Pefa) and dimethyl sulfoxide (DMSO) controls. Error bars are the standard error of the mean. Asterisks denote significant differences between datasets (* = *p* ≤ 0.05, ** = *p* ≤ 0.01). Comparisons that were not significant are labeled “ns”

To elucidate the effects of LSMMG stress on caspase activity, 10 h symbiotic animals were examined with Caspase Glo activity kits (Fig. 8). This time point was chosen as it coincided with the higher expression of caspase transcripts in LSMMG (Fig. 4). Results indicated that LSMMG-treated animals exhibited higher levels of caspase-3/7 and caspase-8 activity compared to gravity conditions in symbiotic animals (Fig. 8a, b). Interestingly, no differences were observed in caspase-9 activity in symbiotic animals incubated under LSMMG or gravity conditions, (Fig. 8c). Executioner activity was observed to be several orders of magnitude higher than both initiator caspases, exhibiting up to a 32-fold increase compared to the initiator caspases in both aposymbiotic and symbiotic animals (Fig. 8a-c). Treatment with the caspase-8 inhibitor Ac-IETD-CHO, the pan-caspase inhibitor Z-VAD-FMK, and the serine protease inhibitor Pefabloc abrogated the significant increase in light organ apoptosis observed at 10 h in LSMMG in symbiotic animals (Fig. 8d).

## Discussion

As humans prepare for space exploration beyond low Earth orbit, it will be critical to fully understand the impact that spaceflight has on mutualistic animal-microbe interactions. Maintaining a healthy microbiome during long-duration spaceflights will require a comprehensive assessment of the effects that environmental stresses, such as microgravity, have on the bacteria-induced development of host tissues [12, 34]. To address this key issue in space biology, we examined the effects of simulated microgravity on the beneficial association between *Euprymna scolopes* and *Vibrio fischeri,* specifically focusing on the phenomenon of bacteria-induced apoptosis in the light organ of the squid host. The outcomes of this study indicated that (i) *E. scolopes* harbors a rich network of genes involved in both extrinsic and intrinsic apoptotic regulation; (ii) the presence of symbiotic bacteria and LSMMG-stress altered the expression of both extrinsic and intrinsic apoptosis gene expression; (iii) the normal, bacteria-induced apoptosis in the squid light organ is mediated in part by caspases that exhibit increased activity under LSMMG; and (iv) the LSMMG-associated increase of caspase activity can be pharmacologically mitigated with inhibitors.

Apoptosis is a critical pathway for animal development playing key roles in tissue homeostasis, antimicrobial defense as well as innate and adaptive immune responses [35, 36]. In recent years, there has been a concerted effort to discern the effects of the microbiome on apoptosis [37, 38] and how this vital form of physiological cell death is altered by microgravity [13, 39]. Although *E. scolopes* has served as a model organism for decades, the pathways by which the symbiont *V. fischeri* induces apoptotic cell death have not been fully delineated with only a few effectors identified [28, 40–42]. The recent sequencing of the *E. scolopes* genome and reference transcriptome has enabled critical animal pathways, including apoptosis, to be investigated [43].

Analyses revealed that *E. scolopes* harbors genes associated with both primary pathways for cell death that are typically seen in animals, including death-receptor mediated extrinsic pathways as well as intracellular stress-induced intrinsic pathways (Fig. 2; Fig. S1; Additional File 1). Results also suggest that *E. scolopes* may form distinct caspase activation platforms, particularly regarding the extrinsic, receptor-mediated, pathway. For example, the absence of Cradd in *E. scolopes*, as well as in the octopus genome, suggests that cephalopod caspase-2 exerts its pro-apoptotic effects outside the PIDDosome complex, likely in association with Tnf receptor signaling [44, 45]. Intriguingly, for 15 apoptosis candidates in the reference transcriptome of *E. scolopes*, no proteins of significant similarity were found in the SwissProt database via BLASTx or BLASTp (Additional File 1)*.* These queries were initially identified based on possessing a CARD- or DED-domain and preliminary BLASTp analysis using non-redundant NCBI database returned mostly uncharacterized and predicted caspase-interacting protein in other cephalopods. Accordingly, these unknown candidates may represent novel apoptotic effectors within the Mollusca phylum.

In general, cephalopods appear to possess a more expansive vertebrate-like repertoire of intrinsic apoptosis genes than other mollusks, including Bnip3, Bax, Apaf1, Htra2, and Pidd [32, 46, 47]. These results suggest that apoptosis has undergone substantial lineage-specific modifications within the Mollusca phylum, which may have resulted in distinct mechanisms of induction. For example, caspase-9 proteins have been described in the mussel *M. galloprovincialis* despite the lack of Apaf1, which is a critical component of the apoptosome complex in other animals [33, 47].

Under the stress of LSMMG conditions, however, there was an acceleration and increased expression of the transcripts associated with bacteria-induced apoptosis genes in symbiotic animals, specifically related to extrinsic, receptor-mediated cell death (Fig. 3). The increased transcriptional response in symbiotic animals was primarily observed among PRR elements for LPS, including LPS-binding proteins Lbp1 and Lbp3, as well as the LPS-induced transcription factor Litaf (Fig. 3). Elevated concentrations of Lbps have been observed in blood plasma of astronauts returning from shuttle missions, as well as the sera of mice following two days of hindlimb suspension [48, 49].

In *E. scolopes*, however, previous work has demonstrated that Lbps1 exhibits a symbiosis-specific function and contributes to bacteria-induced apoptosis in the light organ of *E. scolopes* [28]. Little is known about the functions of Lbp2 and Lbp3 in *E. scolopes* and the precise mechanisms by which symbiotic paralarvae identify LPS remain unclear [50]. However, mammalian Lbps facilitate the receptor-based recognition of gram-negative endotoxin [28], which raises the possibility that the Lbps of *E. scolopes* may be responsible for the delivery of symbiont LPS to death receptors on target host cells, thereby promoting extrinsic apoptosis throughout the ciliated epithelium of the light organ. Additionally, previous studies have shown that LSMMG can increase the shedding of reactogenic LPS by *V. fischeri* [16], thus the combined increase in available MAMP shedding coupled with the increased expression of Lbp1 and Lbp3 receptors in LSMMG may facilitate the accelerated extrinsic apoptosis response in the symbiotic animals under modeled microgravity conditions.

In symbiotic hatchlings, the expression of genes associated with intrinsic, stress-induced, cell death was also increased under LSMMG conditions, specifically within the window of 10 to 12 h post-bacterial colonization (Fig. 3). This increase included the Bh3-only protein Bnip3, the membrane permeabilizer Bak, as well as the mitochondrial proteins Diablo and Aifm3 (Fig. 3). Previous studies with space shuttle-flown murine embryonic stem cells have also shown a similar up-regulation of intrinsic apoptosis-associated genes, including Bnip3 [51].

Bh3-only proteins are the sentinels of stress-induced apoptosis and form the molecular basis of crosstalk between the extrinsic and intrinsic pathways. Bnip3 expression is known to be induced by hypoxia-dependent signaling and reactive oxygen species (ROS), both of which are elements of oxidative stress [52, 53]. Although no evidence of hypoxia was observed in the HARVs during the experimental conditions (Fig. S2), reactive oxygen and nitric oxide stress are critical components during the onset of the squid-vibrio symbiosis [54, 55]. Indeed, the phenomenon of spaceflight and microgravity-induced oxidative stress has been extensively documented in astronauts and many other model organisms and cell lines [23, 56, 57]. Consequently, the up expression of Bnip3, Bak, Diablo, and Aifm3 implies that modeled microgravity-derived oxidative stress may compound the normal, bacteria-induced, development of the light organ in *E. scolopes* via atypical stimulation of the intrinsic pathway.

The results also indicated that the expression of pro-death caspases was increased in symbiotic animals under modeled microgravity. Caspases are cysteine-dependent proteases that are widely regarded as the “core machinery” of apoptosis and are responsible for mediating the initiation and fallout associated with most known pathways for cell death [58]. This study found that both initiator and executioner-type caspases were up-expressed during accelerated light organ apoptosis in modeled microgravity-treated symbiotic hatchlings compared to gravity controls (Fig. 4). The detection of functional caspase enzymes in aposymbiotic controls in the absence of apoptotic cells in the light organ suggests that there is a critical execution threshold for cascade activation. Thus, it appears that the superficial ciliated epithelial cells of *E. scolopes* light organ are “primed” for morphogenesis via baseline levels of initiator and executioner caspase activity that are maintained below the activation threshold for apoptosis until the symbiotic cues for development, such as LPS, are received.

In symbiotic hatchlings, the increase of transcripts encoding pro-death caspases under LSMMG conditions coincided with the early onset and premature peak of developmental cell death (i.e., 6 – 10 h post inoculation) previously observed in the squid [15]. Notably, the amount of initiator caspase has been shown to positively correlate with the extent of downstream executioner activation, and to irreversibly induce apoptosis a certain threshold of activity must be surpassed [59]. These results suggest that the increased caspase expression in LSMMG may lower the activation threshold of light organ apoptosis due to the increased availability of proenzymes, which closely parallels previous reports that the concentrations of pro-caspases-9 and -3 are key predictors of apoptotic susceptibility [60].

The initiators and executioners of *E. scolopes* exhibited a high degree of structural similarity to the orthologs of other animals in particular initiators EsCasp2 and EsCasp8 as well as executioners EsCasp3 and EsCasp7 [61, 62]. However, anomalies were observed in both isoforms of squid caspase-10*,* which contained a single CARD at the N-terminus rather than a dual DED (Fig. 5). This significant deviation from the structural norm indicates that the EsCasp10 sequences were likely misannotated, given that their structure more closely resembles that of initiator caspases-2 and -9 [63].

Additionally, there were deviations in the CASc precursor domain of several caspase isoforms in the host squid, such as EsCasp2_2X and EsCasp9, where the smaller p10 subunit was not observed (Fig. 5). In animals, the primary sequence of the p10 subunit is subject to greater variation than the larger p20 due to the absence of catalytic cysteine and histidine residues, which may have precluded its detection in EsCasp2_2X and EsCasp9. Given that an intact CASc region was identified within these isoforms it is like these two isoforms are functional despite the inability to identify the p10 subunits within the CASC precursor domain.

Maximum likelihood analysis demonstrated that the initiator caspases of *E. scolopes, H. sapiens, R. norvegicus*, *D. rerio*, *X. laevis*, *M. galloprovincialis*, and *O. sinensis* clustered primarily based on like orthologs (Fig. 6). The segregation of initiators into three groups bearing high identity to caspases-8 and -10, -9, and -2 is largely consistent with previous phylogenetic analyses that illustrated further subdivisions between the vertebrates and invertebrates [32, 33]. The exclusion of *E. scolopes* and *M. galloprovincialis* from the subclade of vertebral caspase-9 orthologs, along with their placement on opposing branches of the unrooted phylogenetic tree next to same-species sequences, implies that lineage-specific variations have occurred in the intrinsic initiator caspases of mollusks (Fig. 6).

The increased activity of all tested caspases in symbiotic animals relative to aposymbiotic controls suggests both receptor-mediated and stress-induced apoptosis pathways occurring in the bacteria-induced development of the host light organ (Fig. 7). However, inhibition of caspases under unit gravity resulted in a decrease of only 50% of apoptotic pycnotic nuclei, suggesting that the normal *V. fischeri*-induced apoptosis pathways in the light organ of *E. scolopes* are partially, but not entirely, caspase-dependent. These results align with the previous finding that cathepsin L – a lysosomal, cysteine-dependent protease – also contributes to developmental cell death in symbiotic paralarvae [30], suggesting apoptosis in the host animals is mediated by a multi-faceted array of apoptosis-associated proteases that are triggered by both external and intrinsic signals.

Under the stress of LSMMG, however, our findings suggest that the increase in apoptotic cell death levels in symbiotic light organs is mediated primarily through caspase activation via the extrinsic apoptosis pathway. The phenotype of increased apoptotic nuclei in the light organ under LSMMG was mitigated using caspase inhibitors, producing statistically equivalent numbers of pycnotic nuclei compared to unit gravity controls (Fig. 8d). These increases in caspase activity complement our previous findings that GO terms related to enzymatic activity and catalysis were disproportionately enriched in LSMMG-treated paralarvae at 12 and 24 h compared to gravity controls [23]. These data also parallel previous studies that showed spaceflight and modeled microgravity-induced apoptosis in lymphocytes and erythrocytes was significantly reduced by caspase inhibition [64, 65].

Although most initiator and executioner caspases were up expressed in LSMMG conditions, not all were pharmacologically inhibited. Interestingly, treatment with the Ac-LEHD-CMK caspase-9 inhibitor did not produce a significant reduction in the amount of apoptosis observed under gravity conditions in symbiotic animals (Fig. 7d). Additionally, no LSMMG-induced increase was observed in the caspase-9 activity, despite the increase in transcript levels at 10 h (Fig. 4; Fig. 8). Given that the recognition peptide of the caspase-9 inhibitor is identical to that of the Caspase-Glo substrate (LEHD-CMK), it is unlikely that this phenomenon derives from differences in binding affinity or substrate specificity with the caspase-9 of *E. scolopes*. Caspase-9 has traditionally been considered an upstream caspase that depends on the binding to the CARD motif Apaf-1 apoptosome for activation [46] and is the first caspase of the ROS-regulated mitochondrial apoptotic cascade [66]. Thus, these results suggest there could be alternative pathways for the onset of executioner caspases in the host squid other than caspase-9-dependent pathways. For example, apoptosis can be activated through apoptosome-independent mechanisms [67], thus more research is needed to understand the role of caspase-9 under both gravity and LSMMG conditions.

## Conclusions

Taken together, these results suggest that external stresses, such as modeled microgravity, can cause perturbations to the normal apoptotic pathways in animals. As many of the mechanisms that *E. scolopes* use to communicate with *V. fischeri* are present in other animals (e.g., Lbps) this research provides a framework for understanding how animals respond differentially to the stresses of spaceflight in the presence and absence of their symbionts. For spaceflight, understanding how disruptions in host-microbe homeostasis and animal physiology alter normal physiological responses, such as apoptosis, is critical. Furthermore, the ability to modulate the life or death of a cell as well as identify mitigation targets or strategies to prevent perturbations of animal-microbe homeostasis has immense therapeutic potential for maintaining crew health for long-duration spaceflight.

## Methods

### General procedures

Adult *E. scolopes* were collected from Maunalua Bay on O’ahu, Hawai’I, and transported to the Space Life Sciences Lab where they were maintained in a controlled growth chamber set at 23** °C** on a 12 h light–dark cycle. Egg clutches laid by the female squid were incubated in separate aquaria from the adults for the entirety of paralarvae development (~ 21 days). Newly hatched paralarvae were rinsed twice in 0.22 μm filtered seawater (FSW), then either preserved in an aposymbiotic state (i.e., without *V. fischeri*) or rendered symbiotic by inoculation with the wild-type, strain ES114, to a final concentration of 10^5^ cells per mL of FSW. For all experiments, colonization was confirmed by measuring luminescence with an ATP photometer (GloMax 20/20, Promega Corp., Madison, WI). For symbiotic experiments, log cultures of *V. fischeri* were prepared in seawater tryptone broth [68] and incubated at 28˚C with 225 rpm shaking. For all experiments, animals were anesthetized and euthanized before examination in a 1:1 solution of 0.37 M MgCl_2_ and filtered sterilized seawater. Euthanasia by over anesthesia is the current accepted practice for cephalopods [69, 70].

### Replicates, data treatment, and statistical analyses

All experiments were performed in triplicate, with at least three biological and technical replicates unless otherwise noted. Outliers identified via the 1.5 × interquartile range (IQR) method were removed before analysis, and data normality was assessed via Shapiro–Wilk (SW). Statistical significance was evaluated either using a Welch’s t-test (SW *p* > 0.05) for parametric data or Mann–Whitney-Wilcoxon U test when the data were not normally distributed (SW *p* ≤ 0.05). Both parametric and non-parametric analyses were two-sided and used a significance cut-off of *p* ≤ 0.05.

### Modeled microgravity treatments and DO_2_ readings

To mimic the low-shear fluid conditions of spaceflight, 50-mL High Aspect Ratio Vessels (Fig. 1c; HARVs; Synthecon, Houston, TX) were used as previously described [15]. Briefly, for aposymbiotic conditions, the HARVs were filled with FSW, whereas for symbiotic conditions HARVs contained 105 cells of V. fischeri ES114 per mL of FSW. After animals were added to the HARVs, the reactors were then sealed to prevent the formation of bubbles and incubated at 23 °C in a synchronized LED-illuminated Percival incubator (Percival Scientific Inc., Perry, IA) with rotation at 13 rpm. The HARVs were either rotated around a vertical axis to control for unit gravity (Fig. 1c left) or were rotated around a horizontal axis to mimic microgravity conditions (Fig. 1c right). The dissolved oxygen content (DO2) of the FSW collected from the HARVs was also measured at 24 h with a hand-held probe (Sper Scientific, Scottsdale, AZ) in a minimum of three replicates.

For control purposes, and to establish a baseline for enzyme assays and inhibitor treatments, a cohort of hatchling animals were incubated in borosilicate scintillation vials alongside the HARV reactors until 16 h post-hatching, which marks the peak of developmental cell death in normal gravity [18]. For all treatments, at least one animal from each treatment was put aside to confirm colonization and/or quantify light organ apoptosis. The remaining squid were immediately frozen with liquid nitrogen or dry ice and stored at – 80 °C until downstream analysis.

### RNA isolation, quantification, and quality assessment

For each treatment, RNA was extracted from three dissected light organs in triplicate and pooled using the RNeasy Kit (Qiagen, Valencia, CA) with the previously described modifications [23]. The resultant RNA was quantified using a Qubit 2.0 fluorometer (Life Technologies, Carlsbad, CA) according to the manufacturer’s instructions, and assessments of quality were made using the Agilent 2100 Bioanalyzer (Agilent Technologies, Santa Clara, USA) with the RNA 6000 Nano Kit (Agilent Technologies, Palo Alto, CA). For the nCounter analysis, the RNA samples were diluted to a final concentration of 20 ng per mL of nuclease-free water and stored at – 80 °C.

### Transcriptome analysis and NanoString nCounter

Preliminary gene selection was made by data-mining the reference transcriptome of *E. scolopes* [43] for apoptosis-related KEGG identifiers, GO terms, Pfam accession numbers, and SwissProt annotations (Additional File 1). Target genes were selected for further study by cross-referencing the multi-species KEGG pathway for apoptosis (ko04215) with published analyses in other cephalopods. The selected genes (Table 1; Additional File 2) represent those target genes where probes could be designed. Some apoptosis genes within *E. scolopes* had numerous isoforms and this not all probes were effective. Three LPS-binding proteins (LBPs) from *E. scolopes* were also included in the final apoposis-gene CodeSet (Table 1), along with the housekeeping genes actin (*actB)* and pyruvate carboxylase (*pyc1)*. Probes to the selected target genes were synthesized at NanoString Technologies, Inc. in Seattle, WA. Each assay included eight negative control probes to evaluate the non-specific background and six synthetic RNA targets with matching positive control probes.

### NanoString quality control, data processing, and differential analysis

The raw NanoString counts from the nCounter assay were processed using nSolver software (ver 4.0) as previously described [71]. Quality control was conducted based on imaging quality, probe binding density, positive control linearity, and the limit of detection as established by the negative controls. Non-specific background signal was subtracted, and the data were normalized to the internal controls and housekeeping genes to adjust between technical and biological replicates. Next, the data were imported into R Studio (ver 1.2.1335) and a Shapiro–Wilk test was performed to determine data normality. Afterwards, a LIMMA-voom transformation was applied and the output – expressed as log_2_ counts-per-million (log_2_CPM) – was used to fit a linear regression model. Finally, the differential expression analysis was computed for pair-wise comparisons using non-parametric empirical Bayes statistics with a Benjamin-Hochberg adjustment for multiple testing (Additional File 2).

### Phylogenetic and architectural analysis of caspase enzymes

Caspase protein sequences were obtained from the reference transcriptome for *Euprymna scolopes* [43]*.* Homologous sequences were similarly sourced from the National Center Biotechnology Information (NCBI) for other common research organisms (*Homo sapiens, Rattus norvegicus*, *Xenopus laevis*, *Danio rerio*) as well as two species (*Mytilus galloprovinciali*, and *Octopus sinensis)* within the Mollusca phylum (Additional File 3). To compare initiator and executioner caspases, a multiple sequence alignment was generated via MUSCLE (ver 3.8.31) according to the default parameters [72]. Maximum likelihood analysis was then performed using MEGA X software (ver 10.2.1) assuming the Whelan-Goldman model of amino acid substitution with 1000 bootstrap iterations [73]. The resultant phylogenetic trees were rendered in MEGA X, with branch support values expressed as percentages on the unrooted trees. Separately, the domain architecture of each caspase sequence was analyzed via SMART, InterProScan, and the NCBI Conserved Domain Search [74–76]. Estimates of protein isoelectric point and molecular weight were calculated using the ExPASy Compute pI/Mw tool (Table 2) [77]. The percent identities of the initiator and executioner caspases in *E. scolopes* were determined via Clustal Omega at the default parameters [78].

### Light organ protein isolation and quantification

To examine enzyme activity during light organ apoptosis, total protein was isolated from frozen animals. Per extraction, approximately 60 light organs were dissected into an ice-cold hypotonic extraction buffer containing 24 mM HEPES, 5 mM MgCl_2_, 1 mM EGTA (pH 7.4) supplemented with a custom protease inhibitor cocktail to a final concentration of 2 μg pepstatin, 5 μg aprotinin, 5 μg leupeptin, and 100 μg Pefabloc per mL of buffer. The light organs were then homogenized on ice with a ground-glass micromortar and pestle, and the exudate was centrifuged at 15,000 × g for 15 min at 4 °C to pellet out debris. The amount of protein in the supernatant was subsequently determined using a Qubit 2.0 fluorometer (Life technologies, Carlsbad, CA) according to the manufacturer’s instructions. Afterwards, the protein extract was diluted with ice-cold extraction buffer and 5 M NaCl to a final concentration of 1 mg of protein and 0.45 M NaCl per mL of buffer. All protein extracts were stored at – 80 °C until use. For each treatment, protein extractions were performed in triplicate.

### Caspase activity assays

To quantify caspase activity in the light organ, Caspase-Glo Assay kits (Promega Corp., Madison, WI) were used according to the manufacturer’s instructions with minor modifications. Briefly, equal volumes of pre-warmed Caspase-Glo reagent and light organ protein were combined in a white-walled, flat-bottomed, 96 well plate with 1X protease inhibitor cocktail (Caspase-Glo 3/7) or 60 μM MG-132 proteasome inhibitor (Caspase-Glo 8 and 9) to reduce non-specific signal. The plate was shaken at 400 rpm for 30 s and then transferred to a Synergy microplate reader (BioTek, Winooski, VT) at 25 °C. Luminescence readings were subsequently collected every 2 min for 2 h. Protein samples were prepared in a hypotonic extraction buffer and assayed in duplicate at a final protein concentration of 20 μg per mL of buffer. Duplicate blank reactions consisting of buffer plus proteasome inhibitor were included for each set of samples, and the average was subtracted as background.

### Protease inhibitor treatments

To determine the contributions of different caspases to bacteria-induced apoptosis, hatchling animals were exposed to a series of targeted protease inhibitors. The inhibitors used in this study include the pan-caspase inhibitor z-VAD-FMK, the caspase 8 inhibitor Ac-IETD-CHO, and the caspase 9 inhibitor Ac-LEHD-CMK (Santa Cruz Biotechnology, Dallas, TX) as well as the irreversible serine protease inhibitor, Pefabloc (Sigma Aldrich, St. Louis, MO). All inhibitor solutions were 0.22 µm-filter sterilized and stored at – 80 °C until use. Caspase inhibitors were used at final concentrations of 60 and 100 μM for vial and HARV experiments, respectively. For all experiments, the final concentration of the serine protease inhibitor Pefabloc was 25 μM. These concentrations were chosen in part as they did not cause apparent toxicity to the animal and did not impede colonization. Inhibitor treatment occurred for 2 h before the start of the experiment. Dimethyl sulfoxide (DMSO) controls were run in parallel where appropriate.

### Quantifying apoptotic development in the CEA of the light organ

To visualize and quantify apoptosis in the light organ, hatchling animals were anesthetized and stained in a 1:1 solution of 0.37 MgCl_2_ and FSW containing 0.001% acridine orange dye, which intercalates into the condensed chromatin of dying cells (i.e., pycnotic nuclei) [18]. After staining, a ventral dissection was performed to expose the light organ, and the pycnotic nuclei in each lobe of the light organ were counted via FITC epifluorescence microscopy with a Zeiss Axioplan microscope (Carl Zeiss, Jena, Germany).

## Supplementary Information

**Additional file 1. **

**Additional file 2. **

**Additional file 3.**

**Additional file 4. SupplementalFig. S1.** Summary of the 137 apoptosis genes found in the reference transcriptomeof Euprymna scolopes. Candidates were identified by searching for specific keywords and KEGG identifiers, GO terms, Pfam accession numbers, and SwissProtannotations. The functional and pathway-specific information for each gene wassourced from the top BLASTx hit in the SwissProt database with the lowestE-score.

**Additional file 5. SupplementalFig. S2.** Dissolved oxygen (DO2) content of FSW. Measurements were collectedfrom gravity and LSMMG HARVs following a 24 h incubation period and immediatelyafter 0.22 µm filtration. Data are shown for each colonization phenotype, aswell as the newly filtered seawater. Asterisks denote significant differencesbetween the datasets as determined by Welch’s T-test (* = *p*  ≤ 0.05, ** = *p*  ≤ 0.01). Comparisons that were not significant are labeled “ns”.

**Additional file 6. SupplementalFig. S3.** Apoptotic caspase expression in aposymbiotic hatchlings under gravityand low shear modeled microgravity (LSMMG) conditions. Heatmaps representingthe transcriptional expression of pro-death caspases -2, -3, -8, -9, and -10genes in gravity (left) and LSMMG (right) conditions. Per the color scale, redindicates a negative Z-score and lower-than-average expression, whereas greensignifies a positive Z-score and higher-than-average expression.

**Additional file 7. SupplementalFig. S4.** Identity matrix of initiator and executioner caspases in Euprymnascolopes. Comparison of the amino acid sequence of initiator and executionercaspases (C) in the host squid. Underscore reflects the isoform of the caspase.The scores, expressed as a percent identity (%), have been rounded to thenearest whole number. Numerical scores were determined via Clustal Omega.

**Additional file 8: Table S1.** Transcripts mined from the reference transcriptome of Euprymna scolopes.

**Additional file 9: Table S2.** Caspase homolog sequence information.

## Data Availability

The datasets generated and analyzed in this study are included in this published article and supplemental information files.
