# Supplementary material for: Modeled microgravity alters apoptotic gene expression and caspase activity in the squid-vibrio symbiosis
Source: BMC Microbiol. 2022 Aug 18;22:202. doi: 10.1186/s12866-022-02614-x (PMC9389742; doi:10.1186/s12866-022-02614-x)
Supplement: Supplementary file 4 — Additional file 4. SupplementalFig. S1. Summary of the 137 apoptosis genes found in the reference transcriptomeof Euprymna scolopes. Candidates were identified by searching for specific keywords and KEGG identifiers, GO terms, Pfam accession numbers, and SwissProtannotations. The functional and pathway-specific information for each gene wassourced from the top BLASTx hit in the SwissProt database with the lowestE-score. [file 12866_2022_2614_MOESM4_ESM.pdf]

Function

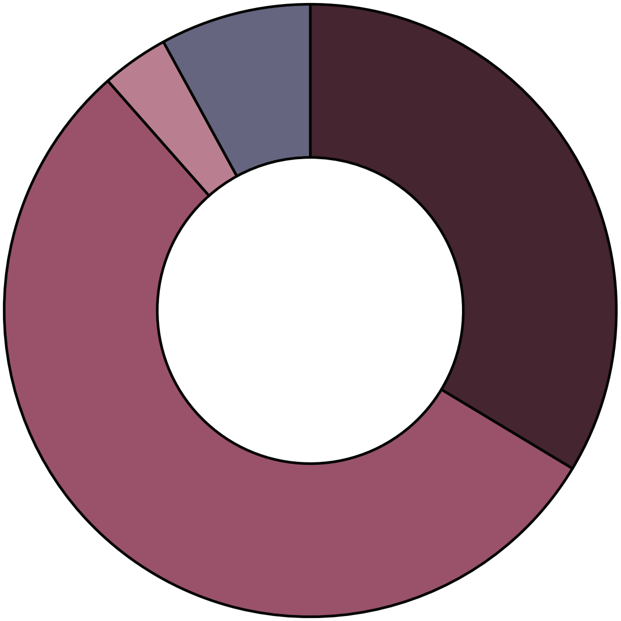

Negative	34%
Positive	55%
Variant	3%
Undetermined	8%

Pathway

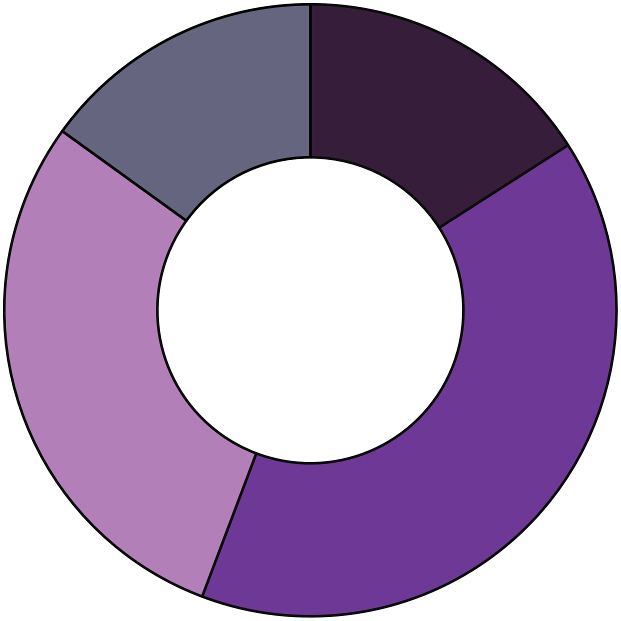

Extrinsic	16%
Intrinsic	40%
General	29%
Undetermined	15%

**Apoptosis-related genes in *Euprymna scolopes***	
Transcripts	293
Unique apoptosis genes	137
