# Supplementary figures and images for: Modeled microgravity alters apoptotic gene expression and caspase activity in the squid-vibrio symbiosis
Source: BMC Microbiol. 2022 Aug 18;22:202. doi: 10.1186/s12866-022-02614-x (PMC9389742; doi:10.1186/s12866-022-02614-x)

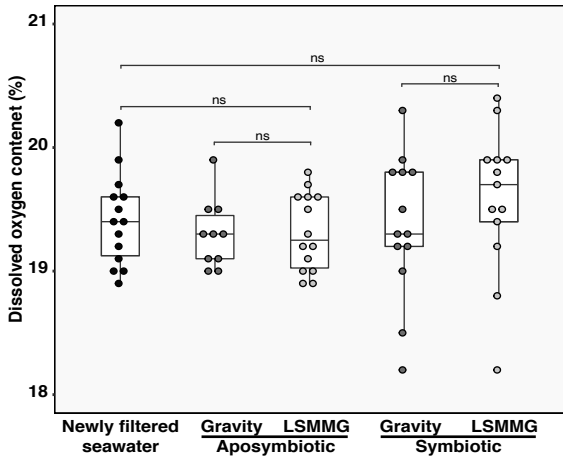

Supplement: Supplementary file 5 — Additional file 5. SupplementalFig. S2. Dissolved oxygen (DO2) content of FSW. Measurements were collectedfrom gravity and LSMMG HARVs following a 24 h incubation period and immediatelyafter 0.22 µm filtration. Data are shown for each colonization phenotype, aswell as the newly filtered seawater. Asterisks denote significant differencesbetween the datasets as determined by Welch’s T-test (* = p ≤ 0.05, ** = p ≤ 0.01). Comparisons that were not significant are labeled “ns”. [file 12866_2022_2614_MOESM5_ESM.pdf]

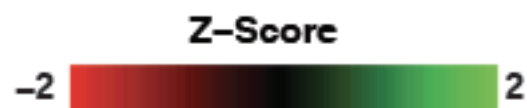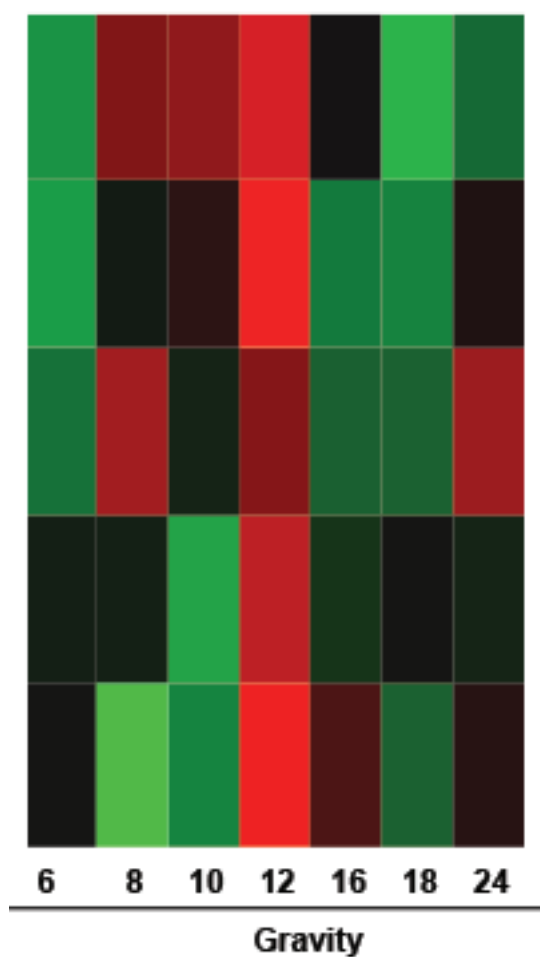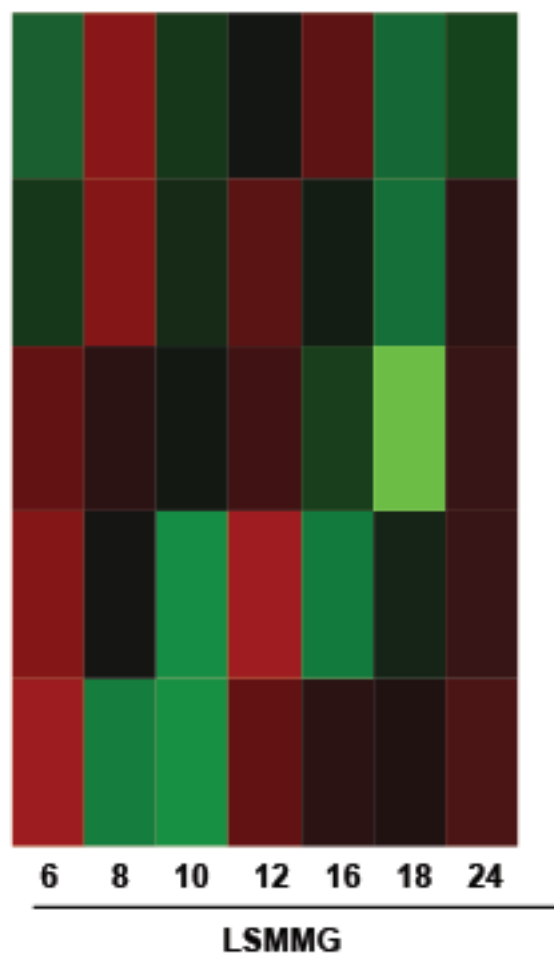

Supplement: Supplementary file 6 — Additional file 6. SupplementalFig. S3. Apoptotic caspase expression in aposymbiotic hatchlings under gravityand low shear modeled microgravity (LSMMG) conditions. Heatmaps representingthe transcriptional expression of pro-death caspases -2, -3, -8, -9, and -10genes in gravity (left) and LSMMG (right) conditions. Per the color scale, redindicates a negative Z-score and lower-than-average expression, whereas greensignifies a positive Z-score and higher-than-average expression. [file 12866_2022_2614_MOESM6_ESM.pdf]
