# Supplementary material for: Modeled microgravity alters apoptotic gene expression and caspase activity in the squid-vibrio symbiosis
Source: BMC Microbiol. 2022 Aug 18;22:202. doi: 10.1186/s12866-022-02614-x (PMC9389742; doi:10.1186/s12866-022-02614-x)
Supplement: Supplementary file 7 — Additional file 7. SupplementalFig. S4. Identity matrix of initiator and executioner caspases in Euprymnascolopes. Comparison of the amino acid sequence of initiator and executionercaspases (C) in the host squid. Underscore reflects the isoform of the caspase.The scores, expressed as a percent identity (%), have been rounded to thenearest whole number. Numerical scores were determined via Clustal Omega. [file 12866_2022_2614_MOESM7_ESM.pdf]

Initiator Caspases

Caspase	C8	C10_2	C10_1	C9	C2_1	C2_2
C8	100					
10_2	29	100				
C10_1	25	95	100			
C9	26	94	95	100		
C2_1	15	16	18	16	100	
C2_2	22	24	22	22	26	100

Executioner Caspases

Caspase	C7_2	C7_4	C3_2	C7_1	C7_3	C3_1
C7_2	100					
C7_4	91	100				
C3_2	35	42	100			
C7_1	34	40	40	100		
C7_3	41	47	49	48	100	
C3_1	37	41	47	55	53	100
