# Supplementary material for: Modeled microgravity alters apoptotic gene expression and caspase activity in the squid-vibrio symbiosis
Source: BMC Microbiol. 2022 Aug 18;22:202. doi: 10.1186/s12866-022-02614-x (PMC9389742; doi:10.1186/s12866-022-02614-x)
Supplement: Supplementary file 9 — Additional file 9: Table S2. Caspase homolog sequence information. [file 12866_2022_2614_MOESM9_ESM.pdf]

Table S2. Caspase homolog sequence information

Initiator*	Accession	Length	Executioner	Accession	Length
HsCasp10	ABJ53426.1	522	HsCasp7	BAG70095.1	336
DrCasp10	AWP39897.1	512	RnCasp7	XP_038945156.1	303
XlCasp10	BAA94751.1	520	DrCasp7	AWP39893.1	350
EsCasp10_X1	g27226.t1	634	XlCasp7	NP_001091272.1	319
EsCasp10_X2	c48267_f2p0_2198	298	MgCasp7	VDI00435.1	410
HsCasp8	XP_005246942.1	523	OsCasp7	XP_029636882.1	283
RnCasp8	XP_006245077.1	482	EsCasp7_X1	g28980.t1	337
DrCasp8	XP_685430.4	520	EsCasp7_X2	c34060_f1p3_1826	371
XlCasp8	NP_001081410.1	520	EsCasp7_X3	TR562630|c0_g1_i1|	297
MgCasp8	VDH92081.1	595	EsCasp7_X4	g36411.t1	213
OsCasp8	XP_029649523.1	502	HsCasp3	AAO25654.1	277
EsCasp8	g57293.t1	560	RnCasp3	NP_037054.1	277
HsCasp2	AAH02427.2	452	DrCasp3	XP_005173133.1	285
RnCasp2	XP_038964243.1	470	XlCasp3	NP_001081225.1	282
DrCasp2	AWP39887.1	437	MgCasp3	VDI74419.1	330
XlCasp2	BAA94746.1	423	OsCasp3	XP_029648145.1	258
MgCasp2	ADZ24778.1	468	EsCasp3_X1	g65085.t1	313
OsCasp2	XP_029647478.1	399	EsCasp3_X2	g19225.t1	365
EsCasp2_X1	g84175.t1	591			
EsCasp2_X2	g71066.t1YRD6	368			
HsCasp9	XP_011540575.1	421			
RnCasp9	AAF85658.1	454			
DrCasp9	NP_001007405.2	436			
XlCasp9	BAA94750.1	399			
MgCasp9	VDH91938.1	505			
EsCasp9	TR95203|c0_g1	408			

*Es, *Euprymna scolopes*; Hs, *Homo* sapiens; Rn, Rattus *norvegicus*; Dr, *Danio rerio*; Xl, *Xenopus laevis*; Mg, *Mytilus galloprovincialis*; Os, *Octopus sinensis*.
